# Genome Sequence of the Extreme Obligate Alkaliphile *Bacillus marmarensis* Strain DSM 21297

**DOI:** 10.1128/genomeA.00967-13

**Published:** 2013-11-27

**Authors:** David G. Wernick, Kwon-Young Choi, Christine A. Tat, Jimmy G. Lafontaine Rivera, James C. Liao

**Affiliations:** Department of Chemical and Biomolecular Engineering, University of California Los Angeles, Los Angeles, California, USAa; Department of Chemistry and Biochemistry, University of California Los Angeles, Los Angeles, California, USAb; The Molecular Biology Institute, University of California Los Angeles, Los Angeles, California, USAc; Institute for Genomics and Proteomics, University of California Los Angeles, Los Angeles, California, USAd

## Abstract

*Bacillus marmarensis* strain DSM 21297 is an extreme obligate alkaliphile able to grow in medium up to pH 12.5. A whole-shotgun strategy and *de novo* assembly led to the generation of a 4-Mbp genome of this strain. The genome features alkaliphilic adaptations and pathways for *n*-butanol and poly(3-hydroxybutyrate) synthesis.

## GENOME ANNOUNCEMENT

*Bacillus marmarensis* strain DSM 21297 is an extreme obligate alkaliphile isolated from mushroom compost near the Marmara region of Turkey (1). *B. marmarensis* has been shown to grow in medium up to pH 12.5 and to possess an extracellular protein and starch-hydrolyzing phenotype (2). This makes *B. marmarensis* an attractive source of biotechnologically and industrially applicable hydrolases. Currently, with a market surpassing $2 billion annually, such alkaline-stable hydrolases have applications in detergents, food additives, and biomass degradation (3). Additionally, only limited genomic information is available for strains that are viable in medium beyond pH 12.0. We report here a draft genome sequence of *B. marmarensis* showing several extracellular hydrolases and biofuel synthesis pathways, and we provide a set of genomic data for the study of extremely alkaliphilic evolution.

*B. marmarensis* genomic DNA was isolated from a culture grown for 24 h at 37°C in alkaline nutrient broth with a Qiagen DNeasy blood and tissue kit according to the manufacturer’s protocol for Gram-positive microbes. The genomic DNA was concentrated by isopropanol precipitation as per standard techniques (4). DNA was sheared and ligated to Illumina adaptors for 100-bp paired-end runs. The sequencing was performed on an Illumina HiSeq 2000 system in the University of California Los Angeles (UCLA) Ely and Edythe Broad Center of Regenerative Medicine and Stem Cell Research High-Throughput Sequencing Core. The sequencing reads were quality filtered using the FASTX toolkit (http://hannonlab.cshl.edu/fastx_toolkit/index.html) and uploaded to the UCLA CNSI Hoffman2 computer cluster for assembly. The assembly was performed using Velvet 1.2.03 (5) with a *k*-mer of 78 bp, a minimum contig length of 200 bp, and a coverage cutoff of 90×. A total of 5.9 million sequence reads were assembled, giving 127-fold coverage of the genome. Genome annotation was performed using both the RAST server (6) and the NCBI GenBank Prokaryotic Genome Automatic Annotation Pipeline (7). The annotation was visualized using Pathway Tools from SRI International (8).

The draft genome consists of 93 large (>500 bp) contigs totaling 4.0 Mb, with a G+C content of 40.2%. A total of 4,195 predicted coding sequences were identified, and 1,889 coding sequences were assigned a predicted function. Among these, 37 tRNA sequences and 7 rRNA clusters were found. Several extracellular hydrolases of industrial importance were annotated: 7 proteases, 6 amylases, 2 cellulases, and 1 lipase. Also, metabolic pathways for the production of the drop-in ready biofuel *n*-butanol (9) and biodegradable plastic poly(3-hydroxybutyrate) (10) were annotated.

Several known adaptations of alkaliphiles were also found in the genome. These include a high number of sodium-proton antiporters (11), sodium-dependent flagellum rotor proteins (12), and a specialized F_1_F_0_-ATPase (13). Interestingly, the F_1_F_0_-ATPase of neutrophilic bacteria contains a GxGxGxG motif in the C subunit that mutates toward AxAxAxA in alkaliphiles; increasing A residues correlate with greater alkaliphilicity (14). However, *B. marmarensis* displays a novel variant of GxSxAxA. This finding, and the rest of the genome, may reveal other unique adaptations necessary for growth in medium beyond pH 12.0.

### Nucleotide sequence and accession numbers.

This whole-genome shotgun project has been deposited at DDBJ/EMBL/GenBank under the accession no. ATAE00000000. The version described in this paper is version ATAE01000000.
